# Effectiveness of Postdischarge Telephone Calls in Reducing Hospital Utilization: Quasi-Randomized Controlled Trial

**DOI:** 10.2196/80529

**Published:** 2026-03-17

**Authors:** Sandie Du, Abigail Anada, Maria Montenegro, Maurie Maitland, Sean Chuan, Sonia Jose, Harveer Sihota, Delia Cooper, Joanna Rivera, Dianne Burditt, Minetaro Naruki-van Velzen, Megan MacPherson

**Affiliations:** 1 Digital Patient and Provider Experience, Fraser Health, Surrey, BC, Canada; 2 Department of Occupation Science & Occupational Therapy, Faculty of Medicine, University of British Columbia, 13737 96th Avenue Office #249, Surrey, BC, V3V 0C6, Canada, 1 6045616605

**Keywords:** patient readmission, hospitalization, emergency room, continuity of care, digital health

## Abstract

**Background:**

Unplanned emergency department (ED) visits and hospital readmissions following discharge contribute to patient distress, increased health care costs, and system inefficiencies. Early postdischarge follow-up can improve care transitions, yet evidence on the effectiveness of telephone-based interventions remains mixed. Telephone calls, a low-barrier form of digital health, may enhance equity and accessibility by reaching patients who face challenges with in-person or higher-technology follow-up.

**Objective:**

This study evaluated the impact of a nurse-led postdischarge telephone intervention delivered by Fraser Health Virtual Care on short-term ED visits and hospital readmissions among recently discharged high-risk patients. Secondary objectives included examining patient experiences with the service and identifying care gaps addressed during follow-up calls.

**Methods:**

A pragmatic quasi-randomized trial was conducted (May 2022-September 2022). Participants were eligible if they were aged 18 years or older and classified as high-risk for readmission using the LACE (Length of stay, Acuity of admission, Comorbidities, and Emergency department use) index (≥10 or <9 and ≥45 y). Participants were allocated to either a postdischarge telephone intervention group or a standard care control group based on daily nurse availability. Intervention participants received a structured nurse-led call 48 hours after discharge assessing understanding of discharge instructions, medication management, follow-up appointments, and home supports. Primary outcomes were ED visits within 7 and 30 days post call; secondary outcomes were hospital readmissions and patient experience. Negative binomial regression models were used to calculate adjusted incident rate ratios (IRRs).

**Results:**

A total of 7091 participants were included (intervention: n=3911, of whom 1752 completed the call; control: n=3180). Postdischarge calls significantly reduced ED visits at both 7 days (adjusted IRR 0.719, 95% CI 0.617‐0.837; *P*<.001) and 30 days (IRR 0.878, 95% CI 0.783‐0.983; *P*=.02). No statistically significant reductions were observed in hospital readmissions at either 7 days (IRR 0.809; *P*=.13) or 30 days (IRR 0.942; *P*=.54). Forty percent of completed calls (n=701) identified at least 1 gap in discharge understanding or follow-up care. Most participants found the calls helpful and reported increased confidence in managing their care.

**Conclusions:**

Structured nurse-led postdischarge telephone calls significantly reduced short-term ED utilization but did not impact readmission rates. These findings support the role of telephone-based virtual care as a scalable, low-barrier strategy to improve care transitions and reduce avoidable ED visits. Additional or ongoing interventions may be required to influence hospital readmission outcomes among high-risk patients.

## Introduction

### Background

High volumes of emergency department (ED) visits and unplanned hospital readmissions following discharge have significant implications for patient outcomes, health care costs, and overall system efficiency. Readmissions within 30 days, which are often considered unplanned and avoidable, are linked to poor patient satisfaction [1], increased mortality risk [2], and substantial financial burden, accounting for over US $2.3 billion annually in Canada [3]. While not all ED visits and readmissions can be prevented, strengthening postdischarge follow-up and care coordination can help reduce these rates and improve patient experience.

The postdischarge period is a particularly vulnerable time for patients as they transition from hospital to home and assume responsibility for their own care. Patients often leave the hospital with new medications, recommended lifestyle changes, or with ongoing care needs, but many may not fully understand their discharge instructions [4]. Limited awareness of their symptoms, difficulty accessing follow-up care, and a lack of support at home can further increase their risk of complications [5]. Without proper guidance and resources, patients are more likely to experience preventable adverse events, leading to ED visits or hospital readmissions [6]. Proactive care coordination during this transition period has the potential to reduce hospital utilization, including ED [7] and hospital readmission rates [8,9]. One promising intervention is postdischarge telephone calls.

### Telephone Calls as Digital Health Interventions

While digital health is often associated with apps, wearables, and video consultations, it is important to recognize that telephone-based care is a legitimate and impactful digital health tool, particularly in real-world contexts where access and equity are paramount. Digital health can be broadly defined as the delivery of care at a distance, the digital storage and sharing of health information, and the use of health-related data to improve services and systems. This broad definition allows inclusion of “low-tech” modalities, such as telephone or SMS text messaging–based interventions, alongside more “high-tech” solutions like app-based programs or algorithm-driven self-management tools [10].

Telephones are widely accessible and require no broadband internet, which makes them particularly valuable for reaching populations who may be marginalized by more complex digital platforms (eg, older adults, those with lower incomes, and those with limited English proficiency) [11]. Unlike video visits and other forms of digital care which demand stable internet and technical know-how [12], telephone calls lower the barrier to entry, enabling broader participation in virtual care [13].

From an equity standpoint, telephone-based care helps mitigate the digital divide [14,15]. Research has shown that disparities in mobile phone ownership are significantly less pronounced than those in internet access or smartphone usage [16]. This positions telephone calls as a uniquely inclusive tool in digital health strategies.

### Postdischarge Calls

Postdischarge calls aim to bridge information gaps, enhance adherence to care plans, and offer emotional support to patients during their transition from hospital to home [17]. Although postdischarge calls have been implemented across various patient populations [18], the evidence supporting their effectiveness remains mixed. Studies have found that postdischarge calls are feasible [19], can improve patients’ experience [20], and may improve follow-up with primary care providers [21]. However, findings on their effectiveness in reducing ED visits and hospital readmissions are inconclusive, partly due to methodological limitations such as small sample size or variability in intervention protocols [22]. Despite these mixed results, the potential for postdischarge calls as part of a comprehensive care intervention continues to be explored, with some studies suggesting their effectiveness in reducing readmissions [23-25].

This study aimed to evaluate the effectiveness of nurse-led postdischarge telephone calls provided by Fraser Health Virtual Care (FHVC) for high-risk patients. Specifically, we examined the impact of a telephone call made 48 hours after hospital discharge on ED visits and hospital readmission within 7 and 30 days following the call. By assessing this intervention, we aim to provide further insight into the role of structured postdischarge follow-up calls in reducing hospital service use and improving patient outcomes.

### FHVC Service

Fraser Health is 1 of 5 health authorities in British Columbia, Canada, and provides care to more than 2 million people across 20 diverse communities through 12 acute care hospitals, outpatient and surgery centers, and various community care sites [26].

In response to the COVID-19 pandemic and the growing need for digitally enabled care, Fraser Health launched FHVC in April 2020, a nurse-led virtual health service that delivers clinical assessment and navigation support through telephone, video, and secure web chat. FHVC services include general health advice and information, personalized clinical assessments, and referrals to appropriate Fraser Health programs and services [26].

FHVC is integrated within the health authority’s digital infrastructure: clinicians document each encounter directly into the electronic health record (EHR), access real-time clinical data, and coordinate follow-up or referrals electronically. The service also supports medical interpreter access, ensuring linguistic inclusivity and equitable reach across the region.

In March 2021, FHVC expanded to include proactive postdischarge outreach calls to patients within 48 hours of hospital discharge. This initiative was introduced as part of a regional digital quality improvement strategy to strengthen care transitions and reduce avoidable ED visits and readmissions. Calls are initiated automatically from daily EHR-generated discharge lists, and nurses follow a standardized digital protocol addressing symptoms, medications, follow-up appointments, and home supports.

This postdischarge component represents a digitally enabled care coordination model that leverages FHVC’s integrated data systems, digital workflows, and virtual communication tools. Unlike usual care, which relies on patients or community providers to initiate follow-up, FHVC’s model provides proactive, system-triggered outreach, a scalable approach to virtual care that blends clinical expertise with health-system data infrastructure.

## Methods

### Study Design

This study was a multicenter, pragmatic quasi-randomized trial conducted across all 12 acute care hospitals in the Fraser Health region (British Columbia, Canada) from May 16, 2022, to September 21, 2022. The trial evaluated the effect of a routine, operationally delivered, postdischarge follow-up phone call on ED visits and hospital readmissions among high-risk patients. The design was pragmatic in nature, embedded within routine care delivery and implemented by the existing FHVC nursing staff, to reflect real-world practice conditions. The trial was retrospectively registered with the ISRCTN registry (registration submission date: September 3, 2025).

The primary objective was to determine the impact of a 1-time follow-up phone call 48 hours after hospital discharge on 30-day ED visits for participants at high risk of readmission. Secondary objectives were to assess 7-day ED visits, 7- and 30-day readmissions, and participant experiences with the service. This trial is reported in accordance with the CONSORT guidelines (Checklist 1).

### Participants and Setting

Participants were adults (≥18 y) discharged from any of Fraser Health’s 12 acute care hospitals. The inclusion and exclusion criteria reflected routine FHVC operational parameters, designed to identify typical patients most likely to benefit from postdischarge follow-up.

Eligibility was informed by the LACE index [9,27,28], a validated measure of 30-day readmission risk incorporating Length of stay (LOS), Acuity of admission, Comorbidities, and Emergency department use. Participants were eligible if they had a LACE score ≥10, or a score <9, and were aged 45 years or older. These thresholds were derived from internal data showing an elevated risk of unplanned readmission.

Exclusion criteria included patients discharged from psychiatric units, to hospice or community nursing programs (where follow-up is routine), those who received additional postdischarge support, or were readmitted or deceased prior to follow-up.

### Intervention

The intervention was a one-time, proactive follow-up call from an FHVC registered nurse approximately 48 hours after discharge. The intervention required no additional infrastructure, using existing FHVC systems, staff, and workflows. Nurses used automated discharge lists generated daily from the EHR to identify eligible participants.

During the calls, nurses followed a semistructured clinical assessment tool covering as follows:

- Understanding of discharge diagnosis and instructions,
- Medication adherence and reconciliation,
- Symptoms or concerns postdischarge, and
- Follow-up appointments and home supports

Calls were tailored to each patient’s discharge plan and medical record. If issues were identified, nurses provided clarification, reinforced education, or facilitated referrals to community or primary care services. Interpreter services were available when required. Nurses used an assessment form to track this information as shown in Appendix S2 in Multimedia Appendix 1.

The intervention reflected usual care processes augmented by proactive outreach and was intentionally flexible to accommodate real-world nursing workflow and patient needs.

### Comparator (Usual Care)

Participants in the control group received standard care only, which typically includes written discharge instructions and follow-up arranged by hospital units or primary care providers at their discretion. No proactive FHVC calls were made to these participants.

### Recruitment and Allocation

Eligible participants were identified through the use of a custom iTracker report that automatically retrieved electronic medical record (EMR) discharge data across Fraser Health hospitals. Each morning, a research assistant applied the eligibility criteria and randomly selected up to 70 participants (using Microsoft Excel’s RAND function) for the intervention group, based on daily nurse capacity. The remaining eligible participants were assigned to the control group.

Because the assignment depended on operational capacity, the allocation was quasi-random and not concealed. On days when the total number of eligible discharges was fewer than 70, all available participants were assigned to the intervention group to maintain operational continuity. Conversely, on days with limited nurse availability, more participants were assigned to the control group. This pragmatic approach preserved ecological validity by embedding group assignment within real-world service delivery constraints.

Registered nurses were blinded to the control group list and conducted intervention calls as part of their routine FHVC workload. Participants were unaware of the research component, ensuring naturalistic behavior.

### Outcomes

Our primary outcome measure was the number of ED visits within 30 days of receiving the intervention, defined as any visit in which a participant registered in any of the 12 EDs within the Fraser Health region, regardless of whether they were ultimately seen by a clinician.

Secondary outcome measures included the following:

1. ED visits within 7 days of the intervention;
2. Hospital readmissions, at 7 and 30 days postintervention, defined as any admission to a hospital postdischarge; and
3. Participant experience, measured via a survey.

### Sample Size Considerations

The sample size calculation was based on a retrospective analysis of EMRs from June 1, 2020, to May 31, 2021. This analysis estimated the average ED visits within 30 days of discharge before and after FHVC’s postdischarge calls were implemented (0.73 and 0.59 ED visits, respectively). To detect a statistically significant difference with 80% power and *α*=.05, a required sample size of 6930 participants (3465 per group) was calculated for 2 independent groups.

### Procedures and Data Collection

FHVC nurses received standardized training to conduct postdischarge follow-up calls from a Clinical Nurse Educator, ensuring consistency in care delivery. Calls were conducted using standard FHVC telehealth systems, and data were recorded in a secure Excel file linked to the EHR.

During each call, FHVC nurses recorded participant information, including name, age, sex, phone number, LACE score, and discharging hospital, into a Microsoft Excel file stored on a secure network drive. Additional data included any identified knowledge gaps during the postdischarge call. A research assistant monitored data daily to ensure completeness and accuracy of data collection.

If participants were unreachable, the nurse left a voicemail message with the FHVC phone number, inviting the participant to call back if they needed support. Data on ED visits, hospital readmissions, LOS of initial hospitalization, city of residence, and the day of discharge were extracted from the EMR using unique identifiers.

All participants were invited via a telephone call to complete a patient experience survey 30 days after the intervention until 80 participants were reached for each of the intervention and control groups. Surveys were administered by a research assistant and a patient partner. The survey instruments differed by study arm to reflect the experience received (see Appendix S3 in Multimedia Appendix 1 for survey questions). Participants who received the postdischarge call from FHVC answered questions about the call itself, including its helpfulness in clarifying discharge instructions, medications, follow-up appointments, and confidence in managing care at home. Participants who did not receive a postdischarge call were asked parallel questions about their discharge experience, including understanding of instructions, medications, follow-up appointments, and confidence in self-management.

### Statistical Analysis

The analysis was completed by a Fraser Health statistician using SPSS (Statistical Package for the Social Sciences) version 21.0 (IBM Corp). The primary analyses followed an intention-to-treat approach. Differences in demographic and outcome variables between groups were analyzed using chi-square tests for categorical variables and the Welch 2-tailed *t* tests for continuous variables. Mann-Whitney *U* tests were used where data were not normally distributed. Average levels of risk factors were compared between groups using relative risks, 95% CIs, and 2-tailed *P* values. To reduce the risk of type I error due to multiple comparisons, a Bonferroni correction was applied to the *P* values from unadjusted univariate tests conducted on demographic and clinical risk variables. This included both between-group comparisons (intervention vs control) and exploratory within-group comparisons (reached vs not reached in the intervention arm). Adjusted *P* values were reported where appropriate.

Negative binomial regression models were used to examine the relationship between the dependent variables (ie, the number of ED visits and count of readmissions at 7 and 30 d post call) and relevant predictors. This modeling approach was selected to account for the overdispersion in the data due to the high occurrence of zero values in the count data for ED visits and hospital admissions. Independent variables included intervention group, age, sex, LACE score, LOS of initial hospitalization, day of discharge of initial hospitalization, and geographical location of ED of initial hospitalization. Regression models were not corrected for multiple comparisons, as they represented prespecified primary analyses. Unlike univariate tests, regression coefficients are estimated jointly, inherently accounting for covariation among predictors. Therefore, corrections for multiple comparisons were not applied.

Incident rate ratios (IRRs) were reported to describe the association between the independent variables and the relative frequency of count of ED visits and count of readmissions at 7 and 30 days. A sensitivity analysis was conducted in which an outlier participant with 23 ED visits was recoded to the next-highest value observed in the dataset (13 visits). The results remained robust following this adjustment. Due to missing values in the initial LOS variable, 32 observations (9 in the control group and 22 in the intervention group) were excluded from the final regression models.

In addition to the primary and secondary outcomes, the number needed to treat (NNT) was calculated to further assess the potential clinical significance of the intervention [29]. NNT is a measure used to determine how many participants need to be treated with the intervention to prevent one additional adverse outcome, such as an ED visit or hospital readmission. The NNT was calculated for the primary outcome of ED visits and the secondary outcome of hospital readmissions at both 7 and 30 days after the follow-up call.

The formula used for calculating NNT is:

*NNT=1/*|*CER* – *EER*|

where CER is the control event rate (ie, the proportion of participants in the control group who experience the adverse outcomes), and EER is the experimental event rate (ie, the proportion of participants in the intervention group who experienced the adverse outcome). The calculated NNT values provide a clinical perspective on the effectiveness of the intervention, helping to assess how many participants need to receive the postdischarge follow-up call to prevent 1 additional adverse outcome.

Descriptive statistics for the participant experience survey were calculated using Microsoft Excel. No inferential tests were conducted because the survey questions differed between groups. The intervention group was asked whether the postdischarge call improved their understanding, and the control group was asked about their understanding of the initial discharge instructions; therefore, direct between-group comparisons were not possible.

### Ethical Considerations

This study was reviewed and approved by the Fraser Health Research Ethics Board (number 2022192). Given that the study evaluated a quality improvement initiative embedded within routine operations and used deidentified secondary administrative data, the requirement for individual informed consent was waived by the ethics committees in accordance with institutional policy. All data were deidentified before analysis. No patient-identifiable information was shared outside the health authority. Data were stored on secure, password-protected servers in compliance with provincial privacy legislation. No financial or material compensation was provided to participants, as all activities represented standard care or evaluation of routine service delivery.

## Results

### Study Population

**Figure 1.**
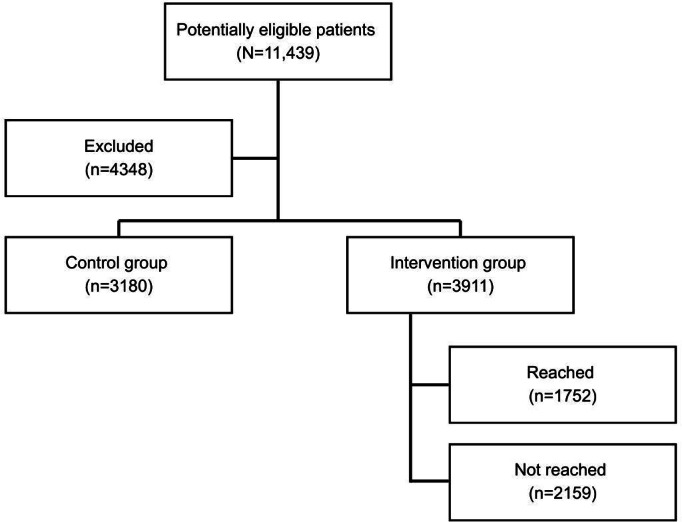
Flow diagram of participant inclusion and study design. This flowchart outlines participant selection for the retrospective cohort study evaluating the impact of nurse-led postdischarge telephone calls provided by Fraser Health Virtual Care (FHVC) on emergency department (ED) visits and hospital readmissions. The study included adult patients (≥18 y) discharged from acute care hospitals within Fraser Health, British Columbia, Canada, between January 2022 and December 2023.

A total of 7091 participants were included in the study, with 3911 in the intervention group and 3180 in the control group (Figure 1). The overall sample had a mean age of 68.1 (SD 13.74) years (95% CI 67.8‐68.4), and 47.2% (n=3349) were female.

Among the participants in the intervention group, 1752 fully completed the postdischarge call, while nurses were unable to reach the remainder directly. Of those who completed the call, 40% (n=701) had at least 1 care gap identified.

### Baseline Characteristics

Baseline characteristics for the intervention and control groups are presented in Table 1. After applying a Bonferroni correction for multiple comparisons (adjusted *α*=.0042 and adjusted *P*<.012), the only statistically significant difference was a higher proportion of weekend discharges in the intervention group (33.8% vs 21.5%; adjusted *P*<.012). No meaningful differences were observed in age, sex, LACE score, LOS, or hospital location. Within the intervention arm, baseline characteristics were similar between participants who completed the call and those who did not (Appendix S4 in Multimedia Appendix 1).

**Table 1.** Baseline characteristics of study sample. Characteristics of patients included in the retrospective cohort study assessing Fraser Health Virtual Care (FHVC)’s postdischarge telephone calls.^a^

	Intervention (n=3911)	Control (n=3180)	Difference statistics	Unadjusted *P* value	Bonferroni adjusted *P* value
Age, mean (SD)	67.7 (13.5)	68.6 (14.1)	6001487.5^b^	.01	.132
Sex, n (%)			*χ*²=7.4^c^	.006	.072
Female	1790 (45.8)	1559 (49)			
Male	2121 (54.2)	1621 (51)			
LACE^d^ score, mean (SD)	8.56 (4.42)	8.29 (4.53)	5943871^b^	.001	.012
LOS^e^ days of initial hospitalization, mean (SD)	5.49 (6.94)	6.57 (9.70)	6001849^b^	.05	.6
Discharge timing, n (%)			*χ*²=131.2^c^	<.001	<.012
Discharged on weekend	1323 (33.8)	684 (21.5)			
Discharged on weekday	2588 (66.2)	2496 (78.5)			
Hospital location, n (%)			*χ*²=3.4^c^	.06	.78
Rural or urban hospital	2278 (58.2)	1783 (56.1)			
Metro hospital	1633 (41.8)	1397 (43.9)			

a Data include demographics, clinical risk factors (eg, LACE score and LOS), discharge timing, and hospital location. Comparisons are presented between the intervention group (received nurse-led call within 48 h postdischarge) and matched control group (no call), with difference statistics and *P* values.

b Mann-Whitney *U *test.

c Chi-square test. All degrees of freedom were 1.

d LACE: Length of stay, Acuity of admission, Comorbidities, and Emergency department use

e LOS: length of stay.

### Primary Outcome: ED Visits

Participants in the intervention group had significantly fewer ED visits at both 7-day and 30-day postdischarge call compared to those in the control group. Within 7 days of the discharge call, 9.33% (365/3911) of intervention participants visited an ED, compared to 11.86% (377/3180) of control participants (Table 2).

The NNT to prevent 1 ED visit within 7 days post call was 40 (95% CI 2774). For ED visits within 30 days, NNT=86; however, the 95% CI crossed zero, indicating nonsignificance.

**Table 2.** Trial outcomes with unadjusted 7-day and 30-day emergency department (ED) visits and hospital readmissions following discharge, and counts and proportions of patients with ED use or hospital readmission after discharge among those who received a postdischarge telephone call (intervention) and those who did not (control) in Fraser Health, British Columbia, Canada (2022–2023).

Postdischarge call	Intervention (n=3911)	Control (n=3180)
Within 7 days of call		
ED visits, n	427	472
ED use, n (%)	365 (9.33)	377 (11.9)
Hospital admissions, n	109	115
Hospital use, n (%)	106 (2.7)	109 (3.4)
Within 30 days of call		
ED visits, n	1072	973
ED use, n	759 (19.4)	654 (20.6)
Hospital admissions, n	292	253
Hospital use, n (%)	257 (6.6)	214 (6.7)

Adjusted analyses confirmed that the intervention was associated with a significant reduction in 7-day ED visits (IRR 0.72, 95% CI 0.62‐0.84; *P*<.001) and a modest reduction at 30 days (IRR 0.88, 95% CI 0.78‐0.98; *P*=.02). Higher LACE scores were consistently associated with increased ED use. No significant associations were observed for age, sex, LOS, day of discharge, or hospital location (Tables3  4).

**Table 3.** Negative binomial regression results for 7-day emergency department (ED) visits following discharge^a^.

	IRR^b^ (95% CI)	*P* value
Intervention	0.719 (0.617-0.837)	<.001
Age (y)	0.996 (0.990-1.001)	.11
Female (vs male)	0.886 (0.762-1.030)	.12
LACE^c^ score	1.051 (1.034-1.068)	<.001
LOS^d^ at initial hospitalization	0.992 (0.982-1.002)	.12
Weekday discharge (vs weekend)	0.982 (0.827-1.164)	.84
Metro hospital (vs rural or urban)	0.897 (0.769-1.045)	.16

a Adjusted IRR and 95% CIs for factors associated with ED visits within 7 days of hospital discharge among adult patients in Fraser Health, British Columbia (2022–2023). The model includes intervention exposure and demographic and clinical covariates.

b IRR: incident rate ratio.

c LACE: Length of stay, Acuity of admission, Comorbidities, and Emergency department use.

d LOS: length of stay.

**Table 4.** Negative binomial regression results for 30-day emergency department (ED) visits following discharge^a^.

	IRR^b^ (95% CI)	*P* value
Intervention	0.878 (0.783‐0.983)	.02
Age	0.991 (0.987‐0.995)	<.001
Female (vs male)	0.941 (0.841‐1.053)	.29
LACE^c^ score	1.082 (1.069‐1.095)	<.001
LOS^d^ at initial hospitalization	1.000 (0.993‐1.006)	.96
Weekday discharge (vs weekend)	0.957 (0.843‐1.086)	.50
Metro hospital (vs rural or urban)	0.897 (0.801‐1.006)	.06

a Adjusted IRR for ED visits within 30 days of hospital discharge among adult patients in Fraser Health, British Columbia (2022–2023), comparing those who received a postdischarge Fraser Health Virtual Care (FHVC) nurse call versus matched controls.

b IRR: incident rate ratio.

c LACE: Length of stay, Acuity of admission, Comorbidities, and Emergency department use.

d LOS: length of stay.

### Secondary Outcome: Hospital Readmissions

There was no statistically significant reduction in hospital readmissions at 7 or 30 days. Within 7 days, 2.7% of intervention participants were readmitted versus 3.4% of controls (IRR 0.81, 95% CI 0.62‐1.06; *P*=.13). At 30 days, readmissions were 6.6% versus 6.7% (IRR 0.94, 95% CI 0.78‐1.14; *P*=.54). LACE score and LOS were significant predictors of readmission, while other covariates were not. NNT estimates to prevent one hospital readmission were 139 (7 days) and 625 (30 days), with 95% CIs crossing zero (Table 2; Appendix S5 in Multimedia Appendix 1).

### Patient Experience Survey Results

Between June 14, 2022, and August 29, 2022, a total of 457 participants (control group: n=223; intervention group: n=264). The study design specified a target sample size of 80 participants per group, and recruitment was concluded once this threshold was reached, resulting in 160 participants (n=80 per group), yielding response rates of 36% in the control group and 30% in the intervention group. Among control group respondents, 67 (83.8%) participants were between the ages of 50 and 70 years, 43 (53.8%) were male, and 51 (63.8%) identified as White. Among intervention group respondents, 56 (70%) participants were between the ages of 50 and 70 years, 45 (56.3%) were male, and 57 (71.3%) identified as White.

Participants in the intervention group reported that the postdischarge call improved their understanding of hospital discharge instructions (n=46, 57.5% agreed) and 40% (n=32) stated that the call provided additional information they did not receive at the time of discharge. Most participants found the postdischarge call helpful in answering their questions (n=33; 41.3% strongly agreed) and 46.3% (n=36) felt more confident managing their own health care after discharge. Nearly all participants viewed the postdischarge call as a valuable service (n=40, 50.6% strongly agreed; n=36, 45.6% agreed). Overall, 71.6% of (n=53) participants in the intervention group visited a primary care provider for follow-up care after discharge.

Among participants in the control group, 47.5% (n=38) strongly agreed that they clearly understood the hospital discharge instructions, and 46.3% (n=37) agreed that they were satisfied with the information provided. Additionally, 51.3% (n=41) felt confident managing their health at home. In total, 60.8% (n=45) of participants in the control group visited a primary care provider for follow-up care after discharge. See Appendix S3 in Multimedia Appendix 1 for full results from patient experience surveys.

## Discussion

### Overview

This study evaluated a 1-time, nurse-led postdischarge follow-up call delivered through an integrated virtual care program. The intervention was associated with modest improvements in early postdischarge outcomes, with participants in the intervention group experiencing significantly fewer ED visits at both 7 and 30 days. However, hospital readmission rates were not significantly affected.

The observed 28% relative reduction in 7-day ED visits and 12% reduction in 30-day ED visits highlight the clinical relevance of the intervention, supported by the NNT estimates which showed that 39 participants needed to receive the intervention to prevent one 7-day ED visit, and 83 patients to prevent one 30-day ED visit. These are clinically meaningful numbers, especially in the context of high hospital discharge volumes, where even modest improvements can translate into substantial system-wide benefits. While hospital readmission reductions were numerically favorable, they did not reach statistical significance, potentially due to lower event rates and limited power. Alternatively, while a postdischarge phone call can clarify instructions, reduce anxiety, and address basic care gaps, a single call may be insufficient to address the complex medical and social factors that contribute to hospital readmissions [30,31] and patients with elevated LACE scores may require more comprehensive or repeated interventions to meaningfully reduce readmissions.

### Patient Experience

Participants receiving postdischarge phone calls reported overall satisfaction with the intervention and found that the calls provided them with additional information, reduced their concerns, and gave them more confidence to manage their own health care at home (Appendix S3 in Multimedia Appendix 1). Interestingly, participants in the control group also reported satisfaction with the discharge information they received, suggesting that unrecognized gaps may remain without structured follow-up. During the postdischarge calls, FHVC nurses asked participants to explain their discharge plan, which often revealed missing or misunderstood information. Nurses followed a standard script which covered essential postdischarge topics. Through this process, 40% of participants in the intervention group were identified as having at least 1 care gap. It is plausible that some participants in the control group had similar gaps, but without prompting, they were not aware of them at the time of the survey, and therefore felt they could manage adequately post discharge. Additionally, 41% of control participants expressed that they would have appreciated FHVC contact information sooner, highlighting the potential value of proactive outreach.

### Potential Mechanisms of Action

Follow-up calls likely operate through multiple mechanisms: reinforcing patient education, supporting medication adherence, addressing emotional concerns, and signaling coordinated care across the health system. The intervention may serve both as a direct support tool and as an organizational signal promoting better discharge and follow-up practices. While single-touch calls improved ED utilization, they appear insufficient to address complex factors driving hospital readmissions, underscoring the need for more intensive or repeated interventions for high-risk populations.

Several studies have demonstrated that patient education [32] and medication adherence [33,34] are critical factors in reducing avoidable health care utilization, which may explain the observed reduction in ED visits in our study. Prior research has demonstrated that inadequate understanding of discharge instructions is a major driver of postdischarge complications and unplanned care use [35,36]. By reinforcing these instructions, postdischarge follow-up calls may have improved patient comprehension of their recovery plan, reduced knowledge gaps, and empowered patients to manage symptoms at home, thereby lowering the need for ED visits. In addition, the calls may have alleviated anxiety and promoted confidence in self-management [37] by providing reassurance and tailored guidance, thereby reducing the likelihood of seeking avoidable emergency care.

Medication adherence is another well-documented challenge following hospital discharge, with nonadherence associated with increased ED visits and readmissions [38]. The follow-up calls offered a valuable opportunity to clarify prescriptions, address concerns, and reinforce adherence strategies. This may have helped prevent adverse events related to medication errors. Similar findings have been observed in previous studies where medication reconciliation and adherence support were associated with reductions in acute care use [32]. These results suggest that postdischarge calls extend beyond simple follow-up; they actively address gaps in understanding, medication adherence, and emotional well-being, contributing to their observed effectiveness in reducing ED visits.

Importantly, within the intervention arm, completing the call did not meaningfully change ED or readmission outcomes compared with participants who were not reached, suggesting that the benefits observed may reflect system-level effects of the outreach model rather than call completion alone. For example, the intervention may have functioned as a signal of coordinated care, reinforcing follow-up workflows, prompting timely triage, or improving discharge planning processes across the health system. Alternatively, exposure misclassification, where a participant assigned to the intervention group did not actually receive a call, may have diluted the measured effect of individual calls, meaning the observed reductions in ED visits reflect the broader impact of the intervention’s integration into routine care rather than direct participant-level engagement alone. Recognizing these mechanisms underscores that single-touch outreach can influence outcomes through organizational processes as well as patient-level interactions.

### Clinical Implications

Postdischarge calls have been shown to enhance patient engagement with primary care providers, improve medication adherence, and contribute to better overall health outcomes [21]. Given the increasing difficulty in accessing primary care [39,40], these calls may serve as a low-barrier intervention to support care transitions and fill gaps in follow-up care.

Discharge calls can facilitate smoother transitions from hospital to home by proactively identifying and addressing common barriers to recovery. By intervening early, the discharge calls can reduce the risk of preventable complications and improve the overall patient experiences [38] while also relieving pressures on overstretched health care systems [41].

Reducing ED visits has important implications for health care systems, as ED overcrowding remains a critical issue [42]. A reduction in ED visits suggests that patients may be managing their conditions more effectively in outpatient settings, potentially leading to cost savings and improved resource allocation. Future research should explore whether postdischarge calls lead to increased engagement with primary care providers, potentially shifting health care utilization patterns.

### Strengths and Limitations

Previous reviews, including 1 Cochrane review [18] and 2 systematic reviews [21,25] have noted that many trials lacked sufficient statistical power, clearly defined sample size calculations, rigorous methodology, or standardized interventions. In contrast, our study addressed these limitations by using a larger sample size, informed by Fraser Health historical data. Additionally, we implemented a structured intervention that included standardized nurse training and call templates, ensuring consistency in care delivery across settings.

A major strength of this trial was its integration into an existing digital health care service. FHVC was already operational before the study, meaning that nurses conducting the calls had prior experience and received training from a clinical nurse educator. This level of standardization may have contributed to the observed effectiveness of the intervention, differentiating our study from others that implemented newly introduced follow-up call programs. Conducted within an existing operational health care service, the study reflected real-world variations in patient care, staffing constraints, and service delivery. As a result, our findings represent an intervention that health care organizations could realistically implement to support patients’ transitions from hospital to home.

The broad eligibility criteria further enhance the generalizability of our findings. Although the study focused on high-risk patients, it included patients with a range of health conditions, demographics, and socioeconomic backgrounds. The standardized discharge call protocol was designed to be adaptable across clinical contexts, and a detailed version is provided for reference (see Appendix S2 in Multimedia Appendix 1).

A key limitation is the potential exclusion of patients without reliable phone access. Certain socioeconomic groups may have faced barriers to participating, including a lack of access to phones or data plans. Although FHVC implemented strategies to enhance accessibility, such as using plain language and integrating interpreters to support patients who do not speak English, these barriers remain a challenge for equitable access to virtual care.

Another limitation of this study was the low intervention exposure rate within the intervention group. While the analysis followed an intention-to-treat approach, a substantial proportion of participants did not answer the postdischarge call and therefore were not actually exposed to the intervention. This may have diluted the observed effect and may have underestimated the true impact of the intervention had more participants engaged. Future efforts should focus on improving patient engagement, such as further increasing awareness of the follow-up service at the point of discharge, to enhance reach and effectiveness.

Despite being guided by a sample size calculation, the study faced logistical challenges due to fluctuating discharge volumes and staffing availability. While the goal was to collect data for 58 days, recruitment extended to 84 days to achieve the desired sample size. Nurse capacity constraints also required adjustments to the intervention group sizes, leading to a control group that was smaller than the initially planned sample size (3180 instead of 3465) and an intervention group that was larger (3911 vs 3465). These adjustments reflect the realities of implementing research within an operational care model and underscore the need to balance methodological rigor with service delivery. These deviations from the original allocation plan may impact statistical power and group comparability and should be considered when interpreting results.

### Future Directions

#### Overview

While this study provides strong evidence for the effectiveness of postdischarge follow-up calls, several areas require further exploration to optimize implementation, improve equity, and maximize impact. Future research should focus on leveraging emerging technologies, understanding the mechanisms driving intervention success, addressing disparities in access to virtual care, and evaluating cost-effectiveness. Additionally, qualitative and subgroup analyses may help further refine the intervention’s design and delivery to better meet the needs of diverse patient populations.

#### Leveraging Emerging Technologies for Enhanced Support

Artificial intelligence (AI) has the potential to improve the scalability and efficiency of postdischarge interventions by augmenting human-led care with data-driven insights. AI-driven decision support systems could help identify patients most likely to benefit from follow-up calls, thereby optimizing resource allocation and ensuring that high-risk individuals receive timely support. By using predictive analytics to prioritize outreach, health care systems could enhance the effectiveness of follow-up programs while also managing staff workload more efficiently.

Additionally, natural language processing technologies could be integrated into call workflows to assist nurses in real-time. These tools could provide structured prompts to ensure comprehensive patient assessments, summarize patient concerns for documentation, or generate automated follow-up messages tailored to each patient’s needs. For example, natural language processing could automatically flag patients with high LACE scores who have missed follow-ups, prompting timely outreach and helping prioritize those at greater risk of admission. Future studies should explore how AI can support, rather than replace, clinical decision-making—maintaining the essential human connection that characterizes high-quality transitional care. A hybrid model where AI improves workflow efficiency while nurses remain the primary point of engagement may offer an optimal balance between scalability and personalized care.

#### Understanding Mechanisms of Action and Patient-Centered Benefits

While this study demonstrated a reduction in ED visits, future research should explore the mechanisms underlying this effect. It remains unclear which components of the intervention, such as patient education, medication adherence support, emotional reassurance, or care coordination, or the simple act of receiving a call, contribute most to the observed improvements. Identifying the most influential elements would support refinement of the intervention to maximize its impact and cost-effectiveness. Involving patients in this refinement process through co-design of the call content or format can further enhance the relevance and effectiveness of the intervention, aligning it more closely with patient needs and preferences.

Beyond clinical outcomes, patient-reported experiences offer valuable insights into the intervention’s effectiveness. Future qualitative research should examine how follow-up calls influence patient confidence in managing their recovery, their perceived quality of care, and any remaining unmet needs following discharge. Additionally, understanding the perspectives of nurses and other health care providers delivering follow-up calls can help identify workflow challenges and opportunities for improvement. Research in this area could inform best practices for scaling and sustaining postdischarge calls while also minimizing provider burden and enhancing patient engagement.

#### Promoting Equity in Postdischarge Support

Ensuring equitable access to follow-up care remains a critical challenge. Patients from lower socioeconomic backgrounds, those with limited English proficiency, and individuals without reliable phone access are at risk of being disproportionately excluded from telephone-based interventions. As a result, those who are most vulnerable to hospital readmission may not receive the support they need. To effectively reach and support diverse populations, follow-up care must also be culturally appropriate and safe. Embedding cultural safety into the design and delivery of interventions can help build trust and improve engagement across communities.

Future research should explore alternative and complementary strategies to reach underserved populations. These may include text-based follow-ups, video consultations, or partnerships with community health workers who can provide in-person support. Additionally, health systems should investigate more seamless integration of interpreter services into virtual care workflows and evaluate the potential of multilingual AI-driven chatbots to help bridge communications barriers. Addressing these disparities is essential to ensure that all patients, regardless of socioeconomic status, digital access, or language, can benefit equitably from postdischarge support.

#### Economic Analysis of Postdischarge Follow-Up Calls

A critical next step is to conduct a cost-effectiveness analysis to determine whether the observed reduction in ED visits results in net cost savings for the health care system. By evaluating factors such as reduced hospital readmissions, decreased ED use, and the cost of nursing time invested in delivering the intervention, an economic evaluation can guide policy decisions and resource allocation for broader implementation.

In addition to understanding overall cost-effectiveness, future studies should assess differential outcomes across patient subgroups. Certain populations may derive more substantial benefit from follow-up calls, informing a more targeted and efficient use of resources. Conducting subgroup analyses based on factors such as age, comorbidities, hospital discharge diagnosis, and social determinants of health could help refine intervention targeting. For example, patients with chronic conditions such as heart failure or chronic obstructive pulmonary disease may benefit more from medication adherence support, while those with lower health literacy may require additional educational reinforcement. Identifying which subgroups experience the greatest reduction in ED visits or readmissions will enable a more tailored approach, ensuring that resources are directed toward those who need them most.

### Conclusions

This study suggests that proactive, digitally enabled outreach may support safer care transitions and reduce early ED utilization, even when delivered as a pragmatic, single-touch intervention within routine clinical operations. The findings highlight the potential for this low-cost, scalable intervention to support safer care transitions, particularly in healthcare systems where timely access to primary care remains a challenge. While the intervention did not significantly reduce hospital readmissions, its impact on ED use underscores its value in improving short-term outcomes and alleviating pressure on acute care services. Future studies should investigate whether certain patient subgroups derive greater benefit and explore ways to improve accessibility for populations with limited phone or digital access.

## Supplementary material

10.2196/80529 Multimedia Appendix 1 Appendix S1: PRECIS-2 assessment of the post-discharge follow-up call trial. Appendix S2: Discharge Call Protocol Checklist. Appendix S3: Patient Experience Survey. Appendix S4: Intervention Group Characteristics (those Reached vs Not Reached). Appendix S5: Negative Binomial regression results for Hospital Readmission,

10.2196/80529 Checklist 1 CONSORT checklist.

## Abbreviations

AI: artificial intelligence
CER: control event rate
ED: emergency department
EER: experimental event rate
EHR: electronic health record
EMR: electronic medical record
FHVC: Fraser Health Virtual Care
IRR: incident rate ratio
LACE: Length of stay, Acuity of admission, Comorbidities, and Emergency department use
LOS: length of stay
NNT: number needed to treat
SPSS: Statistical Package for the Social Sciences
