# Supplementary material for: Effectiveness of Postdischarge Telephone Calls in Reducing Hospital Utilization: Quasi-Randomized Controlled Trial
Source: J Med Internet Res. 2026 Mar 17;28:e80529. doi: 10.2196/80529 (PMC12994759; doi:10.2196/80529)
Supplement: Multimedia Appendix 1 [file jmir-v28-e80529-s001.docx]

**Appendices**

**Appendix S1: PRECIS-2 assessment of the post-discharge follow-up call trial.**

Pragmatic framing of the study design across all PRECIS-2 domains (eligibility, recruitment, setting, organization, delivery flexibility, adherence, follow-up, primary outcome, primary analysis).

| **Domain** | **Pragmatic Justification** |
| --- | --- |
| **Eligibility** | Broad inclusion of adults ≥18 discharged from any Fraser Health hospital who met high-risk criteria (LACE ≥10 or LACE <9 and age ≥45), reflecting patients who would typically receive post-discharge support. Exclusions were limited to populations with alternative support (psychiatric, hospice, community care). |
| **Recruitment** | Participants were identified through automated EMR discharge reports, mirroring real-world hospital operations. No additional recruitment procedures were introduced. |
| **Setting** | Inclusion across 12 acute care hospitals in Fraser Health, representing diverse urban and rural populations and typical hospital workflows. |
| **Organization** | Delivered by existing FHVC nurses as part of routine post-discharge care; no additional staff or resources were required beyond standard operations. |
| **Flexibility: Delivery** | Nurses followed a semi-structured call script but could tailor discussions based on patient EMR information and individual needs, reflecting real-world variability. |
| **Flexibility: Adherence** | Intervention fidelity was maintained through standard training and documentation, but participation was inherently voluntary and based on routine staffing capacity. |
| **Follow-up** | Outcomes (ED visits, readmissions) were captured via EMR, and participant experience surveys were conducted once post-discharge. Minimal researcher interference ensured routine follow-up processes. |
| **Primary Outcome** | ED visits within 30 days, a real-world, clinically meaningful outcome directly relevant to healthcare providers and systems. |
| **Primary Analysis** | Intention-to-treat, unadjusted analysis reflecting all participants as they would experience the service in routine care, with secondary adjusted models for clinical covariates. |

**Appendix S2: Discharge Call Protocol Checklist**

| **Date of Admission:** |  | **Date of Discharge:** | |  | | **Discharge Summary on file:** | ☐ Yes  ☐ No |
| --- | --- | --- | --- | --- | --- | --- | --- |
| **Info provided by:** | ☐ Client  ☐ Other____________  ☐ Both | | **Discharge Diagnosis:** |  | | | |
| **Understanding reason for hospitalization or emergency visit:** | | | | **Comments: N/A= Not applicable** | | | |
| Do you understand the reason for your admission to the hospital? ☐ Yes ☐ No | | | |  | | | |
| Did you receive written instructions when you left the hospital? ☐ Yes ☐ No | | | |  | | | |
| If yes, can you explain it to me?  ☐ Yes ☐ No ☐ N/A | | | |  | | | |
| Were you provided with information on warning signs or what to monitor, and when you would need to seek medical attention? ☐ Yes ☐ No | | | |  | | | |
| **Current Assessment** | | | | | **Intervention** | | |
| **Airway and Breathing** | ☐ Normal Baseline for patient  ☐ Speaking full, clear sentences  ☐ Laboured ☐ Short of breath ☐ Cough  Comments: | | | | ☐ Review clients Home O2 regimen  ☐ Review Medication: ___________  ☐ Seek medical attention Primary care provider/UPCC/WIC  ☐ Emergency Department | | |
| **Circulation** | ☐ No Concerns  ☐ Reports Palpitations ☐ Chest Pain/ Pressure  ☐ Diaphoresis ☐ Dizziness/ light-headedness  ☐ Edema  Comments: | | | | ☐ Education provided____________  ☐ Review Medication: ___________  ☐ Seek medical attention  Primary care provider/UPCC/WIC  ☐ Emergency Department | | |
| **Disability** | ☐ Alert & Orientated/Baseline for patient ☐ Confused  ☐ Sensory/motor changes ☐ Altered Vision  ☐ Pain_____/10 ☐ Blood Glucose ___________  Comments: | | | | ☐ Education provided____________  ☐ Review pain management  ☐ Seek medical attention  Primary care provider/UPCC/WIC  ☐ Emergency Department | | |
| **Surgery**  ☐ N/A | **Surgical Incision:**  ☐ Redness ☐ Swelling  ☐ Drainage ☐ Dressing Dry & Intact  ☐ Pain____/10 ☐ Fever  ☐ Deep breathing & coughing/physio/exercises and mobilize  ☐ Staple/Suture removal in: _________  Comments: | | | | ☐ Review Signs & Symptoms of infection (S/S) (ie: fever/ache/chills, warmth, redness, increased pain, swelling or pus to site)  ☐ Review multimodal analgesia and pain management (ie: alternate, wean)  ☐ Review Deep Vein Thrombosis education (ie: ambulation, watch for skin that is really- red, warm, hurts to touch and/or swollen)  ☐ Seek medical attention Primary care provider/UPCC/WIC  ☐ Emergency Department | | |
| **Gastrointestinal** | ☐ No Concerns ☐ Nausea  ☐ Vomiting ☐ Diarrhea  ☐ Constipation ☐ Bleeding  ☐ Pain____/10 ☐ Inadequate PO intake  LBM:_______________  Comments: | | | | ☐ Review Nausea/vomiting management  ☐ Review constipation prevention  ☐ Review oral rehydration  ☐ Review pain management  ☐ Seek medical attention  Primary care provider/UPCC/WIC  ☐ Emergency Department | | |
| **Genitourinary** | ☐ No Concerns ☐ Dysuria ☐ Frequency  ☐ Urgency ☐ Hematuria ☐ Distention  ☐ Pain____/10 ☐ Difficulty voiding ☐ Catheter  Last Void:______________  Comments: | | | | ☐ Review Pain Management  ☐ Review S/S of infection  ☐ Tips to help urinate  ☐ Trouble shoot catheter  ☐ Seek medical attention Primary care provider/UPCC/WIC  ☐ Emergency Department | | |
| **Musculoskeletal**  ☐ N/A | ☐ No Concerns ☐ Pain: ___/10  ☐ Wound ☐ Swelling ☐ Injury ☐ Other  ☐ Color/Warmth/Movement/Sensation abnormalities  Comments: | | | | ☐ Education provided____________  ☐ Review pain management  ☐ Seek medical attention Primary care provider/UPCC/WIC  ☐ Emergency Department | | |
| 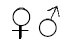  ☐ N/A | ☐ No Concerns  ☐ Bleeding ☐ Discharge ☐ Swelling  ☐ Pain:___/10  Location: ☐ Vaginal ☐ Penile ☐ Scrotal  Comments: | | | | ☐ Education provided____________  ☐ Review pain management  ☐ Seek medical attention Primary care provider/UPCC/WIC  ☐ Emergency Department | | |
| Comments: | | | | | | | |
| **Understanding your medications** | | | | **Comments** | | | |
| Are you on any new medications? ☐ Yes ☐ No | | | |  | | | |
| Have you filled your prescription? ☐ Yes ☐ No | | | |  | | | |
| Do you understand what your medications are and why you are taking them? ☐ Yes ☐ No | | | |  | | | |
| Do you have any questions about your medications?  ☐ Yes ☐ No | | | |  | | | |
| **Follow up appointments** | | | | **Comments** | | | |
| Do you have a follow up appointment booked with your doctor/nurse practitioner or specialist?  ☐ Yes Date:__________________  ☐ No, When will you book? Date:___________________ | | | |  | | | |
| Do you have any other follow up appointments booked (Lab, Imaging, Outpatient clinics)?  ☐ Yes Date:___________________  ☐ No, when will you book? Date:_________________  ☐ N/A | | | |  | | | |
| **Home supports** | | | | **Comments** | | | |
| Since discharge from hospital, are you having trouble moving around or taking care of yourself (ie. dressing, bathing, cooking, and toileting)? ☐ Yes ☐ No  If yes, did you get the recommended equipment/community resources? ☐ Yes ☐ No | | | |  | | | |
| Do you have any support at home if needed?  ☐ Yes ☐ No ☐ N/A  If No, has the client been referred to home health?  ☐ Yes ☐ No | | | |  | | | |
| FHVC contact information given? ☐ Yes ☐ No | | | |  | | | |
| **Advised client to go to ER when:** | | | | | | | |
| ☐ Chest Pain ☐ Shortness of breath ☐ Difficulty breathing ☐ Perfuse Bleeding  ☐ Worsening signs & symptoms of:  Comments: | | | | | | | |
| **Additional Notes:** | | | | | | | |
|  | | | | | | | |

**Appendix S3: Patient Experience Survey**

## Intervention Group *(received a post-discharge call from FHVC)*

| Who completed the survey? | | | ☐ Patient | | | | ☐ Caregiver | | |  | |  | | |  |
| --- | --- | --- | --- | --- | --- | --- | --- | --- | --- | --- | --- | --- | --- | --- | --- |
| The next set of statements are about the call you received from the nurse at Fraser Health Virtual Care after your discharge. Please indicate your level agreement with the following statements: | | | | | | | | | | | | | | | |
| 1 | | The call improved my understanding of the instructions I received when I was discharged from hospital. | ☐ Strongly agree | | | ☒ Agree | | ☐ Disagree | | | ☐ Strongly disagree | | ☐ I don’t know  ☐ Not applicable | | |
| 2 | | The call helped me with a problem I had with *following* the instructions I received when discharged from hospital. | ☐ Strongly agree | | | ☒ Agree | | ☐ Disagree | | | ☐ Strongly disagree | | ☐ I don’t know  ☐ Not applicable/ I did not have any problems | | |
| 3 | | The call improved my understanding of the *follow-up appointments or tests* I needed to complete after my discharge from hospital. | ☐ Strongly agree | | | ☐ Agree | | ☐ Disagree | | | ☐ Strongly disagree | | ☐ I don’t know  ☐ Not applicable | | |
| 4a | | The call improved my understanding of *what* medicines I am taking. 1 | ☐ Strongly agree | | | ☐ Agree | | ☐ Disagree | | | ☐ Strongly disagree | | ☐ I don’t know  ☐ Not applicable | | |
| 4b | | The call improved my understanding of *why* I need to take my medicines. | ☐ Strongly agree | | | ☐ Agree | | ☐ Disagree | | | ☐ Strongly disagree | | ☐ I don’t know  ☐ Not applicable | | |
| 4c | | The call addressed questions and or worries I had about my medicines. | ☐ Strongly agree | | | ☐ Agree | | ☐ Disagree | | | ☐ Strongly disagree | | ☐ I don’t know  ☐ Not applicable | | |
| 5 | | The call provided me with *additional information* that I did not receive when discharged from hospital. | ☐ Strongly agree | | | ☐ Agree | | ☐ Disagree | | | ☐ Strongly disagree | | ☐ I don’t know  ☐ Not applicable | | |
| 6 | | The nurse on the call was helpful in answering my questions. | ☐ Strongly agree | | | ☐ Agree | | ☐ Disagree | | | ☐ Strongly disagree | | ☐ I don’t know  ☐ Not applicable | | |
| 7 | | The call provided me with more confidence to manage my own health care after discharge. | ☐ Strongly agree | | | ☐ Agree | | ☐ Disagree | | | ☐ Strongly disagree | | ☐ I don’t know  ☐ Not applicable | | |
| 8 | | The call reduced concerns I have about managing my own health after discharge. | ☐ Strongly agree | | | ☐ Agree | | ☐ Disagree | | | ☐ Strongly disagree | | ☐ I don’t know  ☐ Not applicable | | |
| 9 | | The call I received from Fraser Health after my discharge from hospital is a valuable service. | ☐ Strongly agree | | | ☐ Agree | | ☐ Disagree | | | ☐ Strongly disagree | | ☐ Not sure | | |
| 10 | | Rate your satisfaction with the overall experience of the call you received. | ☐ Very satisfied | | | ☐ Satisfied | | ☐ Not satisfied | | | ☐ Not very satisfied | | ☐ Not sure | | |
| The next few statements are about your follow-up doctors’ appointments after you left the hospital | | | | | | | | | | | | | | | |
| 11 | | Do you have a family doctor? | ☐ Yes | | | | ☐ No | | | ☐ I don’t know | |  | | |  |
| 12 | | Between leaving the hospital and now, did you see your family doctor for medical care (either in-person or virtually)? (skip to Q3 if answer to Q1 is no) | ☐ Yes | | | | ☐ No | | | ☐ I don’t remember | |  | | |  |
| 13 | | Between leaving the hospital and now, did you visit an Urgent & Primary Care Centre or walk-in clinic to see a doctor or nurse for medical care for something related to the same reason you were hospitalized? This could be either in-person or virtually. | ☐ Yes, please specify | | | | ☐ No | | | ☐ I don’t remember | |  | | |  |
| Demographics (if caregiver, please complete demographics for the patient) | | | | | | | | | | | | | | | |
| 14 | Which city to do you live in? | | | ☐ Abbotsford  ☐ Agassiz  ☐ Burnaby  ☐ Chilliwack | | ☐ Delta North  ☐ Delta South  ☐ Hope  ☐ Langley | | | ☐ Maple Ridge  ☐ Mission  ☐ New Westminster | | ☐ Surrey  ☐ Tri Cities  ☐ White Rock | | | ☐ Other (please specify)  ☐ Prefer not to respond | |
| 15 | What is your age? | | | _____________ (in years) | | ☐ Prefer not to respond | | |  | |  | | |  | |
| 16 | Which of these describe your gender identity? | | | ☐ Male  ☐ Female | | ☐ Transgender  ☐ Non-binary/ non-conforming | | | ☐ Other (please specify) | | ☐ Prefer not to respond | | |  | |
| 17 | Which race or ethnic group best describes you?^2^ | | | ☐ Black  ☐ East Asian  ☐ Southeast Asian | | ☐ Indigenous*  ☐ Latino | | | ☐ Middle Eastern  ☐ South Asian  ☐ White | | ☐ Another race category (please specify) | | | ☐ Do not know  ☐ Prefer not to answer | |
| 18 | *Do you identify as First Nations, Métis and/or Inuk/Inuit? | | | | ☐ First Nations | ☐ Métis | | | ☐ Inuk/Inuit | | ☐ No | | | ☐ Prefer not to answer | |
| 19 | Which of the following best describes your total annual household income before taxes? | | | | ☐ $0  ☐ $1 to $9,999 | ☐ $10,000 to $24,999  ☐ $25,000 to $49,999 | | | ☐ $50,000 to $74,999  ☐ $75,000 to $99,999 | | ☐ $100,000 to $149,999  ☐ $150,000 and greater | | | ☐ Prefer not to respond | |

## Control Group *(did NOT received a post-discharge call from FHVC)*

|  | Who completed the survey? | ☐ Patient | ☐ Caregiver |  |  |  |
| --- | --- | --- | --- | --- | --- | --- |
|  | The next set of statements are about your discharge from the hospital… | | | | | |
| 1 | When I left the hospital, I clearly understood the instructions I received when I was discharged. | ☐ Strongly agree | ☐ Agree | ☐ Disagree | ☐ Strongly disagree | ☐ I don’t know  ☐ Not applicable |
| 2 | After leaving the hospital, I had a problem *following* the instructions I received when discharged from the hospital. | ☐ Strongly agree | ☐ Agree | ☐ Disagree | ☐ Strongly disagree | ☐ I don’t know  ☐ Not applicable |
| 3 | When I left the hospital, I clearly understood the *follow-up appointments or tests* I needed to complete after my discharge from hospital. | ☐ Strongly agree | ☐ Agree | ☐ Disagree | ☐ Strongly disagree | ☐ I don’t know  ☐ Not applicable |
| 4a | When I left the hospital, I clearly understood *what* medicines I am taking.^7^ | ☐ Strongly agree | ☐ Agree | ☐ Disagree | ☐ Strongly disagree | ☐ I don’t know  ☐ Not applicable |
| 4b | When I left the hospital, I clearly understood *why* I need to take my medicines. | ☐ Strongly agree | ☐ Agree | ☐ Disagree | ☐ Strongly disagree | ☐ I don’t know  ☐ Not applicable |
| 4c | When I left the hospital, my questions and or worries I had about my medicines were answered. | ☐ Strongly agree | ☐ Agree | ☐ Disagree | ☐ Strongly disagree | ☐ I don’t know  ☐ Not applicable |
| 5 | When I left the hospital, I was confident that I knew what to do to manage my own health care after discharge. | ☐ Strongly agree | ☐ Agree | ☐ Disagree | ☐ Strongly disagree | ☐ I don’t know |
| 6 | Rate your satisfaction with the information you received during your discharge. | ☐ Very satisfied | ☐ Satisfied | ☐ Not satisfied | ☐ Not very satisfied | ☐ Not sure |
| 7 | Rate your satisfaction with the overall experience of your discharge. | ☐ Very satisfied | ☐ Satisfied | ☐ Not satisfied | ☐ Not very satisfied | ☐ Not sure |
|  | The next few statements are about your follow-up doctors’ appointments… | | | | | |
| 8 | Do you have a family doctor? | ☐ Yes | ☐ No | ☐ I don’t know |  |  |
| 9 | Between leaving the hospital and now, did you see your family doctor for medical care (either in-person or virtually)? (skip to Q10 if answer to Q8 is no) | ☐ Yes | ☐ No | ☐ I don’t remember |  |  |
| 10 | Between now and leaving the hospital, did you visit an Urgent & Primary Care Centre or walk-in clinic to see a doctor or nurse for medical care for something related to the same reason you were hospitalized? This could have been either in-person or virtually. | ☐ Yes, please specify | ☐ No | ☐ I don’t remember |  |  |
|  | Demographics (if caregiver, please complete demographics for the patient) | | | | | |
| 11 | Which city to do you live in? | ☐ Abbotsford  ☐ Agassiz  ☐ Burnaby  ☐ Chilliwack | ☐ Delta North  ☐ Delta South  ☐ Hope  ☐ Langley | ☐ Maple Ridge  ☐ Mission  ☐ New Westminster | ☐ Surrey  ☐ Tri Cities  ☐ White Rock | ☐ Other (please specify)  ☐ Prefer not to respond |
| 12 | What is your age? | _____________ (in years) | ☐ Prefer not to respond |  |  |  |
| 13 | Which of these describe your gender identity? | ☐ Man  ☐ Woman | ☐ Transgender Woman  ☐ Transgender Man | ☐ I prefer to self-describe, please specify: _________ | ☐ Prefer not to respond |  |
| 14 | Which race or ethnic group best describes you?^8^ | ☐ Black  ☐ East Asian  ☐ Southeast Asian | ☐ Indigenous*  ☐ Latino | ☐ Middle Eastern  ☐ South Asian  ☐ White | ☐ Another race category (please specify) ______ | ☐ Do not know  ☐ Prefer not to answer |
| 15 | *Do you identify as First Nations, Métis and/or Inuk/Inuit? | ☐ First Nations | ☐ Métis | ☐ Inuk/Inuit | ☐ No | ☐ Prefer not to answer |
| 16 | Which of the following best describes your total annual household income before taxes? | ☐ $0  ☐ $1 to $9,999 | ☐ $10,000 to $24,999  ☐ $25,000 to $49,999 | ☐ $50,000 to $74,999  ☐ $75,000 to $99,999 | ☐ $100,000 to $149,999  ☐ $150,000 and greater | ☐ Prefer not to respond |

Patient Experience Survey results – Intervention Group

| **#** | **Survey question** | **Strongly agree** | **Agree** | **Disagree** | **Strongly disagree** | **I don’t know** | **Not applicable** |
| --- | --- | --- | --- | --- | --- | --- | --- |
| 1 | The call improved my understanding of the instructions I received when I was sent home from the hospital. | 25  (31.3%) | 46  (57.5%) | 4  (5.0%) | 2  (2.5%) | 3  (3.8%) | 0  (0%) |
| 2 | The call helped me with a problem I had with following the instructions I received when I was sent home from the hospital. | 10  (12.5%) | 36  (45.0%) | 14  (17.5%) | 1  (1.3%) | 8  (10.0%) | 11  (14%) |
| 3 | The call improved my understanding of the follow-up appointments or tests I needed to complete after I was sent home from hospital. | 13  (16.3%) | 46  (57.5%) | 10  (12.5%) | 1  (1.3%) | 6  (7.5%) | 4  (5.0%) |
| 4a | The call improved my understanding of what medicines I am taking. | 12  (15.0%) | 35  (43.8%) | 15  (18.8%) | 1  (1.3%) | 5  (6.3%) | 12  (15.0%) |
| 4b | The call improved my understanding of why I need to take my medicines. | 14  (17.5%) | 30  (37.5%) | 16  (20.0%) | 1  (1.3%) | 6  (7.5%) | 13  (16.3%) |
| 4c | The call addressed questions and or worries I had about my medicines. | 13  (16.3%) | 12  (15.0%) | 20  (25.0%) | 1  (1.3%) | 3  (3.8%) | 20  (25.0%) |
| 5 | The call provided me with additional information that I did not receive when I was sent home from the hospital | 11  (13.8%) | 32  (40.0%) | 31  (38.8%) | 1  (1.3%) | 4  (5.0%) | 1  (1.3%) |
| 6 | The nurse on the call was helpful in answering my questions. | 33  (41.3%) | 33  (41.3%) | 3  (3.8%) | 3  (3.8%) | 1  (1.3%) | 7  (8.8%) |
| 7 | The call provided me with more confidence to manage my own health care after I was sent home from the hospital | 23  (28.8%) | 37  (46.3%) | 10  (12.5%) | 1  (1.3%) | 5  (6.3%) | 4  (5.0%) |
| 8 | The call reduced concerns I have about managing my own health after being sent home from the hospital | 18  (22.5%) | 48  (60.0%) | 7  (8.8%) | 1  (1.3%) | 2  (2.5%) | 4  (5.0%) |
| 9 | The call I received from Fraser Health after I was sent home from hospital is a valuable service. | 40  (50.6%) | 36  (45.6%) | 2  (2.5%) | 0  (0.0%) | 1  (1.3%) | 0  (0.0%) |
|  |  | **Very satisfied** | **Satisfied** | **Not satisfied** | **Very not satisfied** | **I don’t know** |  |
| 10 | Rate your satisfaction with the information you received during your discharge. | 43  (53.8%) | 37  (46.3%) | 0  (0%) | 0  (0%) | 0  (0%) |  |
|  |  | **Yes** | **No** |  |  |  |  |
| 11a | Do you have a family doctor? | 74  (92.5%) | 6  (7.5%) |  |  |  |  |
| 11b | If yes, between leaving the hospital and now, did you see your family doctor for medical care? | 53  (71.6%) | 21  (28.4%) |  |  |  |  |
| 11c | If no, between leaving the hospital and now, did you visit an Urgent & Primary Care Centre or walk-in clinic to see a doctor or nurse for medical care? | 2  (33.3%) | 4  (66.7%) |  |  |  |  |

Patient Experience Survey Results – Control Group

| **#** | **Survey question** | **Strongly agree** | **Agree** | **Disagree** | **Strongly disagree** | **I don’t know** | **Not applicable** |
| --- | --- | --- | --- | --- | --- | --- | --- |
| 1 | When I left the hospital, I clearly understood the instructions I received when I was discharged. | 38  (47.5%) | 30  (37.5%) | 7  (8.8%) | 4  (5.0%) | 0  (0.0%) | 1  (1.3%) |
| 2 | After leaving the hospital, I had a problem following the instructions I received when discharged from the hospital. | 5  (6.3%) | 6  (7.5%) | 42  (52.5%) | 22  (27.5%) | 3  (3.8%) | 2  (2.5%) |
| 3 | When I left the hospital, I clearly understood the follow-up appointments or tests I needed to complete after my discharge from hospital. | 28  (35.0%) | 37  (46.3%) | 4  (5.0%) | 3  (3.8%) | 2  (2.5%) | 6  (7.5%) |
| 4a | When I left the hospital, I clearly understood what medicines I am taking. | 32  (40.0%) | 30  (37.5%) | 5  (6.3%) | 4  (5.0%) | 0  (0%) | 9  (11.3%) |
| 4b | When I left the hospital, I clearly understood why I need to take my medicines. | 33  (41.3%) | 32  (40.0%) | 2  (2.5%) | 4  (5.0%) | 0  (0%) | 9  (11.3%) |
| 4c | When I left the hospital, all my questions and or worries I had about my medicines were addressed | 27  (33.8%) | 34  (42.5%) | 5  (6.3%) | 2  (2.5%) | 2  (2.5%) | 10  (12.5%) |
| 5 | When I left the hospital, I was confident that I knew what to do to manage my own health care after discharge. | 27  (33.8%) | 41  (51.3%) | 6  (7.5%) | 5  (6.3%) | 1  (1.3%) | 0  (0%) |
|  |  | **Very satisfied** | **Satisfied** | **Not satisfied** | **Very not satisfied** | **I don’t know** |  |
| 6 | Rate your satisfaction with the information you received during your discharge. | 33  (41.3%) | 37  (46.3%) | 6  (7.5%) | 2  (2.5%) | 2  (2.5%) |  |
| 7 | Rate your satisfaction with the overall experience of your discharge. | 32  (40.0%) | 34  (42.5%) | 8  (10.0%) | 6  (7.5%) | 0  (0%) |  |
|  |  | **Yes** | **No** |  |  |  |  |
| 8a | Do you have a family doctor? | 74  (92.5%) | 6  (7.5%) |  |  |  |  |
| 8b | If yes, between now and leaving the hospital, did you see your family doctor for medical care? | 45  (60.8%) | 29  (39.2%) |  |  |  |  |
| 8c | If no, between now and leaving the hospital, did you visit an Urgent & Primary Care Centre or walk-in clinic to see a doctor or nurse for medical care? | 4  (66.7%) | 2  (33.3%) |  |  |  |  |

**Appendix S4: Intervention Group Characteristics (those Reached vs Not Reached)**

Characteristics of patients included in the retrospective cohort study assessing FHVC’s post-discharge telephone calls intervention group split by those who received vs those who did not receive the intervention (i.e., a voicemail was left). Data include demographics, clinical risk factors (e.g., LACE score, length of stay), discharge timing, and hospital location. Comparisons are presented between the intervention group (received nurse-led call within 48 hours post-discharge) and matched control group (no call), with difference statistics and p-values

|  | **Intervention, Call completed (*N=* 1,752)** | **Intervention, Call not completed (*N=2,159)*** | **Difference statistics and p values** | **Unadjusted P Value** | **Bonferroni adjusted P** |
| --- | --- | --- | --- | --- | --- |
| **Age, *mean (SD)*** | 68.5 (12.8) | 67.1 (13.9) | U= 17779224 | 0.0011 | 0.0132 |
| **Female, *n* (%)** | 785 (45.0%) | 1005 (46.5%) | Χ²= 1.18 | 0.29 | 1.00 |
| ***Male, n (%)*** | 967 (55.0%) | 1154 (53.5) |  |  |  |
| **LACE score, Mean (SD)** | 8.43 (4.43) | 8.67 (4.41) | U= 1823574 | 0.053 | 0.64 |
| **LOS days of initial hospitalization, Mean (SD)** | 5.35 (6.71) | 5.60 (7.13) | U= 1836455 | 0.32 | 1.00 |
| **Day of the week of initial discharge, *n* (%)** |  |  |  |  |  |
| **Weekend** | 613 (34.99%) | 710 (32.89%) | Χ²= 1.91 | 0.17 | 1.00 |
| **Weekday** | 1139 (65.01%) | 1449 (67.11%) |  |  |  |
| **Hospital location, n (%)** |  |  |  |  |  |
| **Rural/Urban** | 1025 (58.50%) | 1241 (57.48%) | Χ²= 0.57 | 0.45 | 1.00 |
| **Metro** | 720 (41.10%) | 913 (42.29%) |  |  |  |

Trial outcomes. Unadjusted 7-day and 30-day emergency department (ED) visits and hospital readmissions following discharge. Counts and proportions of patients with ED use or hospital readmission after discharge among those who received a post-discharge telephone call (intervention) and those who did not (control) in Fraser Health, British Columbia, Canada (2022–2023).

|  | Intervention, Call completed (*N=* 1,752) | Intervention, Call not completed (*N=2,159)* |
| --- | --- | --- |
| *Within 7 days of call* |  |  |
| ED visits | 206 | 211 |
| ED use | 176 (10.05%) | 189 (8.75%) |
| *Hospital admissions* | 52 | 57 |
| *Hospital use* | 52 (2.97%) | 54 (2.50%) |
| Within 30 days of call |  |  |
| ED visits | 471 | 601 |
| ED use | 362 (20.66%) | 397 (18.38) |
| *Hospital admissions* | 129 | 163 |
| *Hospital use* | 117 (6.68%) | 140 (6.48%) |

**Appendix S5: Negative Binomial regression results for Hospital Readmission**

Negative binomial regression results for 7-day hospital readmission following discharge. Modelled associations between post-discharge FHVC telephone calls and readmission risk within 7 days of discharge, adjusted for demographics, LACE score, and hospital characteristics. Fraser Health, BC (2022–2023).

|  | IRR | CI | P-value |
| --- | --- | --- | --- |
| Intervention | 0.809 | 0.616 - 1.063 | 0.128 |
| Age | 1.009 | 0.999 - 1.019 | 0.085 |
| Female (vs Male) | 1.143 | 0.874 - 1.500 | 0.327 |
| LACE score | 1.086 | 1.058 - 1.113 | 0.000 |
| LOS at initial hospitalization | 1.012 | 0.999 - 1.021 | 0.039 |
| Weekday discharge (vs weekend) | 1.029 | 0.753 - 1.387 | 0.855 |
| Metro hospital (vs rural/urban) | 1.084 | 0.827 - 1.418 | 0.556 |

Negative binomial regression results for 30-day hospital readmission following discharge. Results from multivariable negative binomial regression assessing 30-day readmission risk after discharge for patients who received FHVC nurse-led calls versus controls in Fraser Health, BC (2022–2023).

|  | IRR | CI | P-value |
| --- | --- | --- | --- |
| Intervention | 0.942 | 0.779 - 1.140 | 0.536 |
| Age | 1.006 | 0.999 - 1.012 | 0.107 |
| Female (vs Male) | 1.148 | 0.952 - 1.385 | 0.147 |
| LACE score | 1.116 | 1.095 - 1.138 | 0.000 |
| LOS at initial hospitalization | 1.014 | 1.005 - 1.023 | 0.000 |
| Weekday discharge (vs weekend) | 0.992 | 0.799 - 1.225 | 0.938 |
| Metro hospital (vs rural/urban) | 0.973 | 0.804 - 1.175 | 0.774 |

**Appendix S6: CONSORT Checklist of items for reporting pragmatic trials**

| Section | Item | Standard CONSORT description | Extension for pragmatic trials | Completed (Y/N) |
| --- | --- | --- | --- | --- |
| Title and abstract | 1 | How participants were allocated to interventions (eg, “random allocation,” “randomised,” or “randomly assigned”) |  | Y |
| **Introduction** |  |  |  |  |
| Background | 2 | Scientific background and explanation of rationale | Describe the health or health service problem that the intervention is intended to address and other interventions that may commonly be aimed at this problem | Y |
| **Methods** |  |  |  |  |
| Participants | 3 | Eligibility criteria for participants; settings and locations where the data were collected | Eligibility criteria should be explicitly framed to show the degree to which they include typical participants and/or, where applicable, typical providers (eg, nurses), institutions (eg, hospitals), communities (or localities eg, towns) and settings of care (eg, different healthcare financing systems) | Y |
| Interventions | 4 | Precise details of the interventions intended for each group and how and when they were actually administered | Describe extra resources added to (or resources removed from) usual settings in order to implement intervention. Indicate if efforts were made to standardise the intervention or if the intervention and its delivery were allowed to vary between participants, practitioners, or study sites | Y |
|  |  |  | Describe the comparator in similar detail to the intervention |  |
| Objectives | 5 | Specific objectives and hypotheses |  | Y |
| Outcomes | 6 | Clearly defined primary and secondary outcome measures and, when applicable, any methods used to enhance the quality of measurements (eg, multiple observations, training of assessors) | Explain why the chosen outcomes and, when relevant, the length of follow-up are considered important to those who will use the results of the trial | Y |
| Sample size | 7 | How sample size was determined; explanation of any interim analyses and stopping rules when applicable | If calculated using the smallest difference considered important by the target decision maker audience (the minimally important difference) then report where this difference was obtained | Y |
| Randomisation—sequence generation | 8 | Method used to generate the random allocation sequence, including details of any restriction (eg, blocking, stratification) |  | Y |
| Randomisation—allocation concealment | 9 | Method used to implement the random allocation sequence (eg, numbered containers or central telephone), clarifying whether the sequence was concealed until interventions were assigned |  | Y |
| Randomisation—implementation | 10 | Who generated the allocation sequence, who enrolled participants, and who assigned participants to their groups |  | Y |
| Blinding (masking) | 11 | Whether participants, those administering the interventions, and those assessing the outcomes were blinded to group assignment | If blinding was not done, or was not possible, explain why | Y |
| Statistical methods | 12 | Statistical methods used to compare groups for primary outcomes; methods for additional analyses, such as subgroup analyses and adjusted analyses |  | Y |
| **Results** |  |  |  |  |
| Participant flow | 13 | Flow of participants through each stage (a diagram is strongly recommended)—specifically, for each group, report the numbers of participants randomly assigned, receiving intended treatment, completing the study protocol, and analysed for the primary outcome; describe deviations from planned study protocol, together with reasons | The number of participants or units approached to take part in the trial, the number which were eligible, and reasons for non-participation should be reported | Y |
| Recruitment | 14 | Dates defining the periods of recruitment and follow-up |  | Y |
| Baseline data | 15 | Baseline demographic and clinical characteristics of each group |  | Y |
| Numbers analysed | 16 | Number of participants (denominator) in each group included in each analysis and whether analysis was by “intention-to-treat”; state the results in absolute numbers when feasible (eg, 10/20, not 50%) |  | Y |
| Outcomes and estimation | 17 | For each primary and secondary outcome, a summary of results for each group and the estimated effect size and its precision (eg, 95% CI) |  | Y |
| Ancillary analyses | 18 | Address multiplicity by reporting any other analyses performed, including subgroup analyses and adjusted analyses, indicating which are prespecified and which are exploratory |  | Y |
| Adverse events | 19 | All important adverse events or side effects in each intervention group |  | N/A |
| **Discussion** |  |  |  |  |
| Interpretation | 20 | Interpretation of the results, taking into account study hypotheses, sources of potential bias or imprecision, and the dangers associated with multiplicity of analyses and outcomes |  | Y |
| Generalisability | 21 | Generalisability (external validity) of the trial findings | Describe key aspects of the setting which determined the trial results. Discuss possible differences in other settings where clinical traditions, health service organisation, staffing, or resources may vary from those of the trial | Y |
| Overall evidence | 22 | General interpretation of the results in the context of current evidence |  | Y |

***Cite as:*** *Zwarenstein M, Treweek S, Gagnier JJ, Altman DG, Tunis S, Haynes B, Oxman AD, Moher D for the CONSORT and Pragmatic Trials in Healthcare (Practihc) group. Improving the reporting of pragmatic trials: an extension of the CONSORT statement. BMJ 2008; 337;a2390.*
